# Episodic Canopy Structural Transformations and Biological Invasion in a Hawaiian Forest

**DOI:** 10.3389/fpls.2017.01256

**Published:** 2017-07-21

**Authors:** Christopher S. Balzotti, Gregory P. Asner

**Affiliations:** Department of Global Ecology, Carnegie Institution for Science, Stanford CA, United States

**Keywords:** carnegie airborne observatory, forest change, forest gaps, invasive species, lidar, remote sensing

## Abstract

The remaining native forests on the Hawaiian Islands have been recognized as threatened by changing climate, increasing insect outbreak, new deadly pathogens, and growing populations of canopy structure-altering invasive species. The objective of this study was to assess long-term, net changes to upper canopy structure in sub-montane forests on the eastern slope of Mauna Kea volcano, Hawai‘i, in the context of continuing climate events, insect outbreaks, and biological invasion. We used high-resolution multi-temporal Light Detection and Ranging (LiDAR) data to quantify near-decadal net changes in forest canopy height and gap distributions at a critical transition between alien invaded lowland and native sub-montane forest at the end of a recent drought and host-specific insect (*Scotorythra paludicola*) outbreak. We found that sub-montane forests have experienced a net loss in average canopy height, and therefore structure and aboveground carbon stock. Additionally, where invasive alien tree species co-dominate with native trees, the upper canopy structure became more homogeneous. Tracking the loss of forest canopy height and spatial variation with airborne LiDAR is a cost-effective way to monitor forest canopy health, and to track and quantify ecological impacts of invasive species through space and time.

## Introduction

Forests are inherently dynamic systems that experience episodic mortality. However, recent work has shown that global forests are undergoing accelerated mortality due to climatological factors (Allen et al., 2010). In many forests, larger canopy trees are thought to be at higher risk due to greater exposure to solar radiation, biotic agents, and increased susceptibility to xylem cavitation (e.g., Nepstad et al., 2007; Floyd et al., 2009). A consequence of this disproportionate risk is a potential structural shift at the scale of whole landscapes to forests dominated by smaller trees (Phillips et al., 2010). A large-scale shift in forest structure would alter crucial ecosystem services, such as carbon sequestration, nutrient cycling, water storage, and future climate regulation (Phillips et al., 2009; Anderegg et al., 2013).

With or without climate change, other factors can selectively alter forest structure, such as invasive species, insect outbreaks, and tree pathogens (Dale et al., 2001). These disturbance agents can drive localized or landscape-scale shifts in species composition and structure, mediated through pest-pathogen and host interactions (Holdenrieder et al., 2004). Such compositional shifts can lead to changes in forest structure and function at multiple spatial and temporal scales, and provide opportunities for establishment or expansion of invasive species (Dukes et al., 2009; Boyd et al., 2013). Although much invasive species research has focused on losses of biodiversity, invasive species can also transform the three-dimensional structure and light utilization of forests (Asner et al., 2008). Invasive plant species alter forest structure in a multitude of ways, including replacement of native canopy species, rapid infilling of gap space, alteration of nutrient availability, and modification of prevailing disturbance regimes (Ellison et al., 2005; Hughes and Denslow, 2005; Asner et al., 2008).

The Hawaiian Islands have been recognized as threatened by changing climate (Loope and Giambelluca, 1998), increasing insect outbreaks (Banko et al., 2014), new deadly pathogens (Keith et al., 2015), and growing populations of stand-altering invasive species (Asner et al., 2008). With roughly half of the plants in Hawai‘i classified as non-native (Wagner et al., 1999), these threats can generate changes in forest structure with cascading effects on critically imperiled understory species that have evolved to exist under native canopy architecture. It has been well documented that low elevation wet forests in Hawai‘i have been severely modified by past human use (Hughes and Denslow, 2005). These forests have also been heavily invaded by alien tree species that have altered the canopy structure of the once native forests (Hughes and Denslow, 2005; Asner et al., 2008). Most of Hawai‘i’s remaining native forests persist only in upslope refugia, spanning elevations from sub-montane to treeline ecological zones, above the lowland forests dominated by alien canopy species. We do not, however, know the degree to which these remaining native forests have changed in recent times, or the rate at which alien forest species have expanded (or not) upslope into remaining native forests.

Our ability to quantify and track canopy changes at ecologically meaningful scales across landscapes requires high-resolution remote sensing approaches, including specialized sensors, such as small footprint Light Detection and Ranging (LiDAR; Zimble et al., 2003). With the proven effectiveness and increased availability of LiDAR over the last two decades, high-resolution landscape-scale studies of canopy structure and gap dynamics have become feasible in Hawaiian forests (e.g., Kellner and Asner, 2009). However, much of the work with small footprint LiDAR in general, has been from single time periods or short intervals (<5 years.). Little is known about how forest structure has been changing over longer periods of time at these same scales (Marinelli et al., 2016). The objective of this study was to assess long-term, net changes in forest structure of a Hawaiian sub-montane forest, in the context of continuing climate events, insect outbreaks, and biological invasion. In particular, we used a multi-temporal LiDAR data set over our study landscape from before and after a major insect outbreak and drought to quantify near-decadal net changes in forest canopy height, canopy height variation, and gap distributions at a critical transition between alien invaded lowland and native sub-montane forest. We then discuss potential mechanisms for the observed changes in forest structure between Hawaiian invaded lowlands and sub-montane forests. This transition zone has undergone moderate drought starting in 2008 (Frazier and Giambelluca, 2016; Supplementary Figures S1, S2), followed by a major insect (koa moth; *Scotorythra paludicola*) outbreak that heavily impacted one of the two dominant native canopy trees, koa (*Acacia koa*), throughout the region in 2013 (Banko et al., 2014).

## Materials and Methods

### Study Region

**FIGURE 1:**
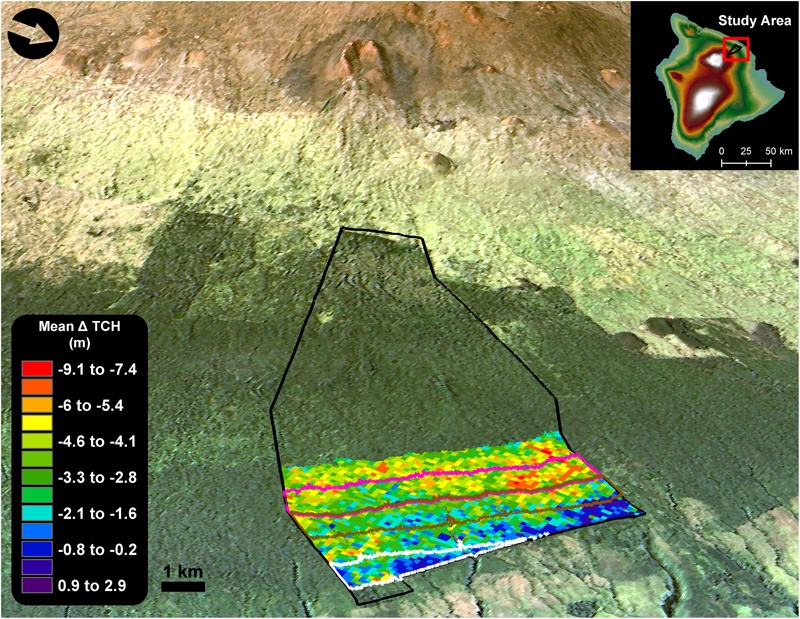
Change in top-of-canopy height (TCH) in forests of the Laupāhoehoe study region (600–1100 m a.s.l.), located on the eastern slope of Mauna Kea volcano, Hawai‘i island (155°15′54.63″W, 19°56′11.13″N). The black outline is the extent of the Laupāhoehoe forest reserve. Areas in red represent the greatest amount of canopy height loss between 2007 and 2016. The lower forests outlined in white (600–700 m a.s.l.) are dominated by invasive tree species that were established well before 2007(established-invasion). Forests outlined in brown (800–900 m a.s.l.) are in the process of invasion by alien tree species (invasion-outbreak), and the native trees have experienced a defoliation event from a koa moth (*Scotorythra paludicola*) outbreak in 2013. Upper forests in pink (900–1000 m a.s.l.) are native with minimal presence of alien tree species (native-outbreak); however, they were also heavily defoliated by the 2013 koa moth outbreak.

The study region, hereafter referred to as Laupāhoehoe, was made up of portions of two adjoining forest reserves, located on the eastern slope of Mauna Kea volcano, Hawai‘i (**Figure 1**). Laupāhoehoe is an important watershed that provides habitat for a minimum of 16 endangered plant species, five endangered birds, and Hawai‘i’s only native land mammal, the ‘ōpe‘ape‘a (Hawaiian hoary bat; *Lasiurus semotus*; Department of Land and Natural Resources [DLNR] and United States Department of Agriculture [USDA], 2016). The Laupāhoehoe reserve is part of the largest remaining native dominated forests in Hawai‘i and ranges in elevation from roughly 518–1860 m above sea level (a.s.l.). The lower portions (<900 m) of Laupāhoehoe canopy have been highly altered by alien invasive tree species, predominantly *Psidium cattleianum* and *Ficus rubiginosa* (Asner et al., 2009; Broadbent et al., 2014; Department of Land and Natural Resources [DLNR] and United States Department of Agriculture [USDA], 2016). This invasion is not unique to Laupāhoehoe and is representative of the existing conditions between highly invaded lowland wet and native sub-montane forests throughout Hawai‘i (Hughes and Denslow, 2005; Zimmerman et al., 2008). The focus of this study was on the 1,572 ha transition zone between the highly invaded lower elevation forest and the native sub-montane forests (600–1000 m a.s.l.). The upper canopy of the native sub-montane forest was dominated by the two most widespread native tree species in Hawai‘i, koa and ‘ōhi‘a (*Metrosideros polymorpha*). The native forests also had a well-developed sub-canopy layer made up of tree ferns (*Cibotium glaucum, C. chamissoi*, and *C. hawaiiense*) and other native tree species (*Myrsine lessertiana, Coprosma rhynchocarpa*, and *C. pubens*). In highly invaded forests, the native sub-canopy layer was lacking, often replaced by thick monotypic stands of *P. cattleianum* (Asner et al., 2008; Department of Land and Natural Resources [DLNR] and United States Department of Agriculture [USDA], 2016).

Our focal landscape was broken into three forest classes by elevation, based on the degree of invasion by alien tree species, and a koa moth defoliation (**Table 1**). Elevation transects by Jones (2011) revealed that in Laupahoehoe, *P. cattleianum* stem density has a strong linear trend (*r*^2^ = 0.78) with elevation up to roughly 1000 m. *P. cattleianum* decreases with increasing elevation. The lower portions of the reserve (<800 m) had *P. cattleianum* stem densities > 6500 stems ha^-1^. Stem density dropped to 130 stems ha^-1^ by 950 m, and no *P. cattleianum* stems were recorded at 970 m (**Table 1** and Supplementary Table S1). The native koa tree density has an opposite trend, decreasing with decreasing elevation. Scowcroft and Sakai (1984) found that the lower portions of koa stands in Laupahoehoe start at roughly 750 m, and contained 840 koa trees (diameter > 1.3 cm) ha^-1^. The average number of koa trees slowly increased with elevation until the density more than tripled between 890 m (820 trees ha^-1^) and 920 m (2840 trees ha^-1^; Supplementary Table S2). As a result of the largest recorded koa moth outbreak, mature koa trees found between 800 and 2000 m a.s.l in Laupāhoehoe experienced multiple defoliations between 2013 and 2014, resulting in widespread dieback and mortality (Banko et al., 2014). The endemic koa moth is a koa specialist, with previous outbreaks causing as much as 35% mortality of mature defoliated trees. The three classes used in this study were: established-invasion, invasion-outbreak, and native-outbreak. Established-invasion was made up of forests found between 600 and 700 m that had previously been documented as heavily invaded by *P. cattleianum* and *F. rubiginosa* from well before 2007 (Asner et al., 2009). Invasion-outbreak forests were found between 800 and 900 m, within the zone of the 2013–2014 koa moth outbreak (800–2000 m). Additionally, invasion-outbreak forests were invaded by the same alien tree species as the established-invasion forests (Department of Land and Natural Resources [DLNR] and United States Department of Agriculture [USDA], 2016), but to a lesser degree (Jones, 2011). Native-outbreak forests were found between 900 and 1000 m., contained the most aboveground carbon stocks, and had a minimal presence of invasive tree species (**Table 1**). However, native koa trees in the native-outbreak forest were also heavily defoliated by the recent koa moth outbreak.

**Table 1:** Study landscapes analyzed within Laupāhoehoe sub-montane forests.

Study landscape	Analysis area (ha)	Mean elevation (m a.s.l.)	MAP (mm)	MAT (°C)	P. *cattleianum* density ha^-1∗^	*A. koa* density ha^-1†^	*Scotorythra* peak abundance (moths trapped per day)^‡^
Established-invasion	174.5	662	4958	18.2	nd	nd	nd
Invasion-outbreak	443.8	850	4559	17.2	3324	883	(20–30)
Native-outbreak	504.3	950	4372	16.6	398	2520	(120–130)

*^∗^Data from Jones (2011), ^†^Data from Scowcroft and Sakai (1984), ^‡^Data from Banko et al. (2014). Mean annual precipitation (MAP) and mean annual temperature (MAT) data obtained from http://climate.geography.hawaii.edu/ (Giambelluca et al., 2013, 2014). Aboveground carbon density (ACD) data calculated from the top-of-canopy height based on Asner et al. (2016).*

### LiDAR Data Collection

Small footprint airborne LiDAR data were collected over the study region in January 2007 and 2016 from ∼2000 m a.s.l. by the Carnegie Airborne Observatory (CAO). In 2007, Laupāhoehoe was sampled with the CAO-Alpha configuration described in detail in Asner et al. (2007, 2008), resulting in a mean return density of 2.6 m^-2^. In 2016, the upgraded CAO Airborne Taxonomic Mapping System (CAO-AToMS), described in Asner et al. (2012), was used with a mean return density of 2.5 m^-2^. In short, LiDAR data were combined with an embedded Global Positioning System-Inertial Measurement Unit (GPS-IMU) to determine the 3-D location of each laser return from the sensor, creating a “point cloud” of the laser return data. The resulting geolocated points were then interpolated into raster digital terrain models (DTM) for the ground surfaces, and digital surface models (DSM) for the canopy. Subtraction of the DTM from the DSM produced canopy height models (hereafter referred to as top-of-canopy height; TCH), at a mapping resolution of 1.25 m for 2007 and 1 m for 2016. The DTM models were interpolated from triangular irregular network (TIN) models, created from the ground points. The DSM models were interpolated from TIN models, fit to first returns. In 2016, ground returns were classified using the Lasground program, which is part of the Lastools suite (Rapidlasso, GmbH; Gilching, Germany). The spatial error of the CAO LiDAR system was determined to be <0.15 m vertically and <0.36 m horizontally (Asner et al., 2010). Validation studies of the CAO-derived TCH, across a wide range of studies, including Hawai‘i, have shown these approaches to be highly accurate (e.g., Asner et al., 2008; Taylor et al., 2015), with vertical errors in Hawaii tree heights reported between 0.5 and 0.9 m (Asner et al., 2008). Because the 2007 data were slightly more coarse with a mapping resolution of 1.25 m, we resampled both years to a spatial resolution of 2 m for this study, focusing the study on changes greater than 2 m × 2 m.

### Canopy Height Change

**FIGURE 2:**
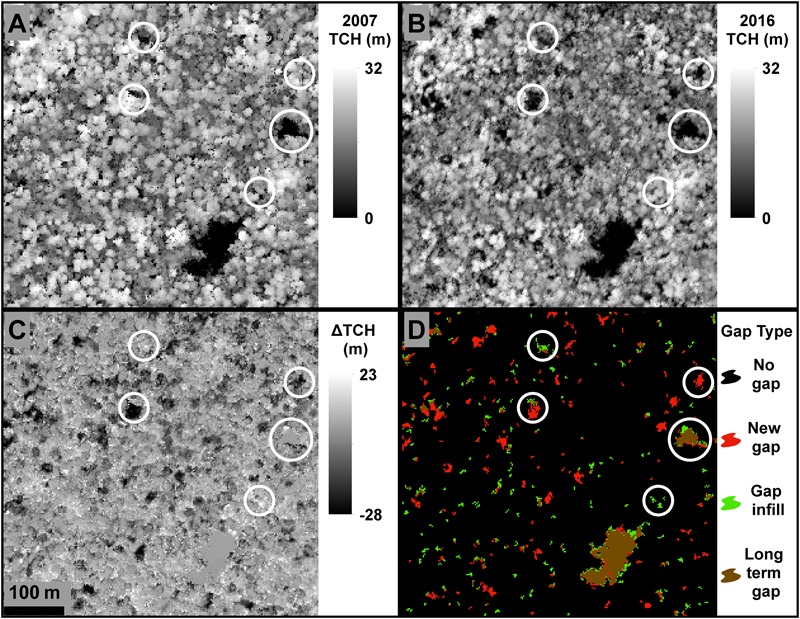
Example results on changes in top-of-canopy height (TCH) and gaps, derived from airborne LiDAR. **(A)** TCH in 2007. **(B)** TCH in 2016. **(C)** Change in TCH between 2007 and 2016. **(D)** Gap dynamics from 2007 to 2016, showing newly formed gaps in red, filled gaps in green, and gaps that have persisted from before 2007 in brown.

Once the LiDAR data had been converted to TCH layers, we determined the amount of change in TCH by subtracting the 2007 TCH layer from the 2016 TCH layer (**Figure 2**). Density distributions were created for each forest type to quantify and visualize the net change in TCH over the past 9 years. Within each forest type, we also calculated the TCH coefficient of variation (CV; Eq. 1) for each year to compare the change in forest canopy structure variation between years and forest types.

$$\text{CV=}(\frac{\text{SD}}{\bar{\text{X}}})\;\text{*}\;\text{100}$$

From the TCH layers, we also calculated the aboveground carbon density for each forest type (ACD; Mg C ha^-1^) using ArcGIS (ESRI 2011. ArcGIS Desktop: Release 10.2.1 Redlands, CA: Environmental Systems Research Institute) and a Hawaii specific TCH to ACD allometric equation developed by Asner and Mascaro (2014) and Asner et al. (2016).

$$\text{ACD=3}.\text{744}\;\text{*}\;{\text{TCH}}^{\text{1}.\text{391}}$$

We then calculated the median change in TCH and the CV across the study landscape within 1ha tiles. These 1 ha tile calculations were done in order to identify regions within the forest that showed a decrease in TCH and a loss of variation (i.e., forests that are becoming shorter and more homogeneous in upper canopy structure, the invasion front).

For further analysis of the distribution of change within each forest landscape, TCH data (at the 2 m mapping resolution) were classified into five height classes (minimal change ± 2 m, loss of 2–10 m, loss > 10 m, gain of 2–10 m, gain > 10 m). Minimal change of ±2 m was used as a conservative estimate for minor changes, and potential error in the LiDAR instruments. The estimated error for the LiDAR-derived TCH for 2007 was <0.7 ± 2 m (Asner et al., 2008). Loss and gains between 2 and 10 m represented changes to tree canopies (dieback and growth), but not total loss or addition of new upper canopy structure. Loss and gains > 10 m represented a substantial change to the upper canopy structure of the forests. Change >10 m was chosen to represent loss or gain of upper canopy, based on past research in the Laupāhoehoe area that found most mid-canopy species reach heights from 4.8 to 10 m depending on the forest type (Asner et al., 2008). A chi-squared analysis was applied to determine the statistical relevance of the observed frequency of TCH change by height class between forest types. A random sample of 1000 points per forest type (*n* = 3000) was used for the chi-squared (χ^2^) analysis with a minimum distance of 10 m between samples, to reduce the probability of sampling two points from one tree crown.

### Gap Distributions

Canopy gaps were defined here as contiguous areas of forest canopy below the dominant canopy height (Hunter et al., 2015), more specifically in this study as areas >12 m^2^ in size, and 70% below the mean canopy height of the surrounding 1 ha forest as described in Marvin and Asner (2016). The Marvin and Asner (2016) method was implemented in ArcGIS to define gaps based on structural dynamics of local forests, accounting for variables such as height of the forest and recently fallen trees on their sides. For each year, we created a mean TCH layer (TCH_m_) using a 1 ha smoothing kernel. The TCH_m_ layer was then subtracted from the original TCH layer and divided by the TCH_m_, resulting in a relative TCH layer. Each year’s relative TCH was used to determine the location of canopy openings. Static gaps were defined as relative TCH > -0.7, or >70% below the mean canopy height of the surrounding 1 ha forest. To reduce false positives, gaps with an area of <12 m^2^ (<3 contiguous pixels) were removed (Marvin and Asner, 2016). The static gap layers for 2007 and 2016 were used to classify the forest into four gap classes: no gaps, new gaps, gap infill, and long-term gaps (**Figure 2** and **Table 2**). No gaps were forested areas that did not experience a static gap in 2007 or 2016. New gaps (dynamic gaps) were gaps that were present in 2016 but not in 2007. Gap infills were areas within gaps that were classified as static gaps in 2007, but were no longer classified as gaps in 2016. Long-term gaps were areas classified as a static gap in both years. A chi-squared test was applied to determine statistical relevance of the observed patterns between forest types. A random sample of 1000 points per forest type (*n* = 3000) was also used for the chi-squared (χ^2^) test with a minimum distance of 10 m between samples, to reduce the probability of sampling two points from one gap. All post processing statistical analyses were carried out in R (R Development Core Team, 2016), ENVI (ITT Visual Information Solutions: Release 4.8 Boulder, CO, United States), and ArcGIS.

**Table 2:** Gap classifications used.

Classification	Gap in 2007	Gap in 2016
No Gap	–	–
New Gap	–	x
Gap Infill	x	–
Long-term Gap	x	x

## Results

**FIGURE 3:**
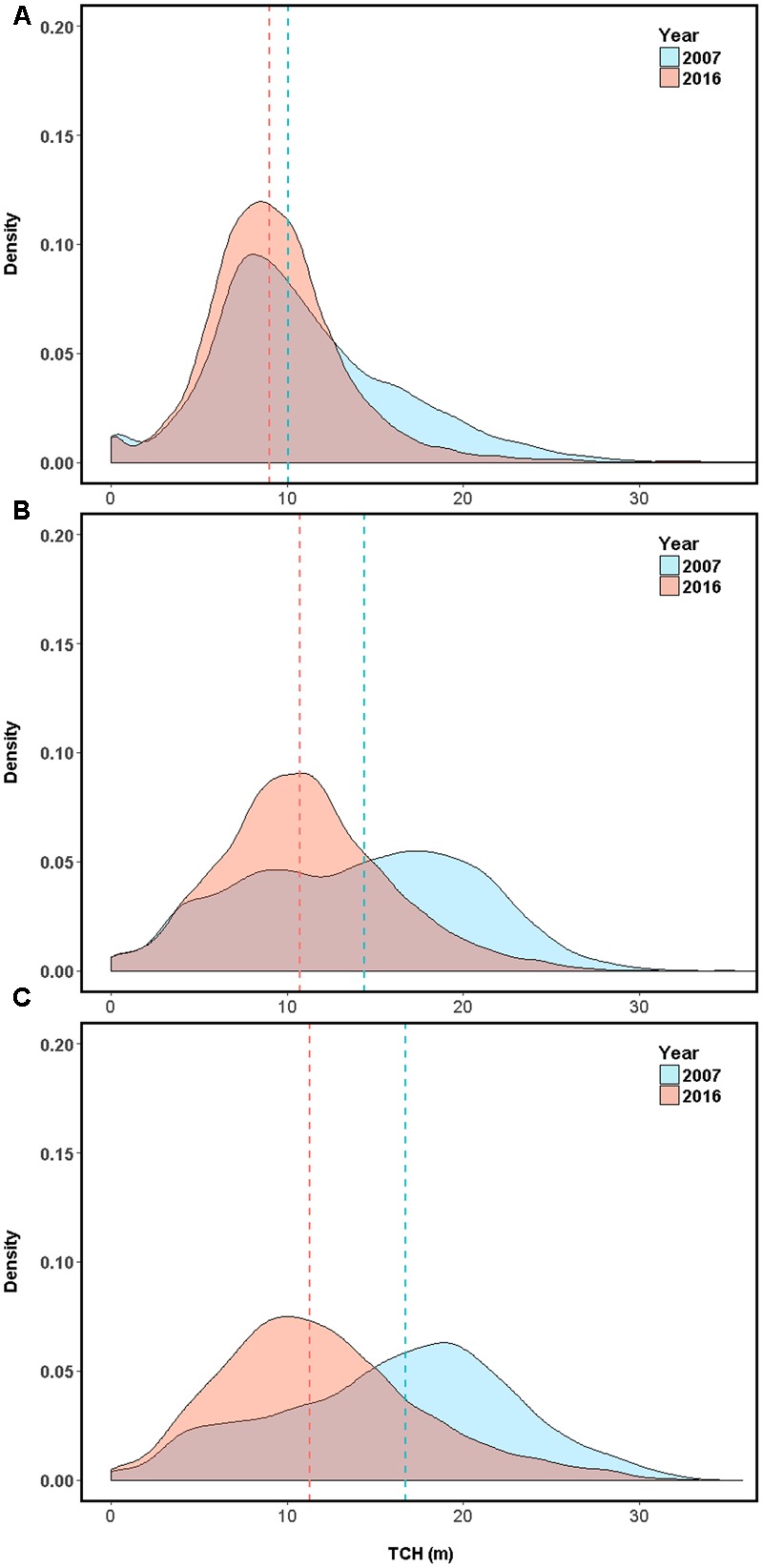
Top-of-canopy height (TCH) distribution on the eastern slope of Mouna Kea volcano, Hawaii (155°15′54.63″W, 19°56′11.13″N) for **(A)** established tree invasion area dominated by alien *Psidium cattleianum* and *Ficus rubiginosa* (established-invasion) **(B)** tree invasion plus koa moth (*Scotorythra paludicola*) outbreak (invasion-outbreak). **(C)** Koa moth outbreak with minimal tree invasion (native-outbreak). Median values for each year are represented with dashed lines, with 2007 in blue and 2016 in red.

**FIGURE 4:**
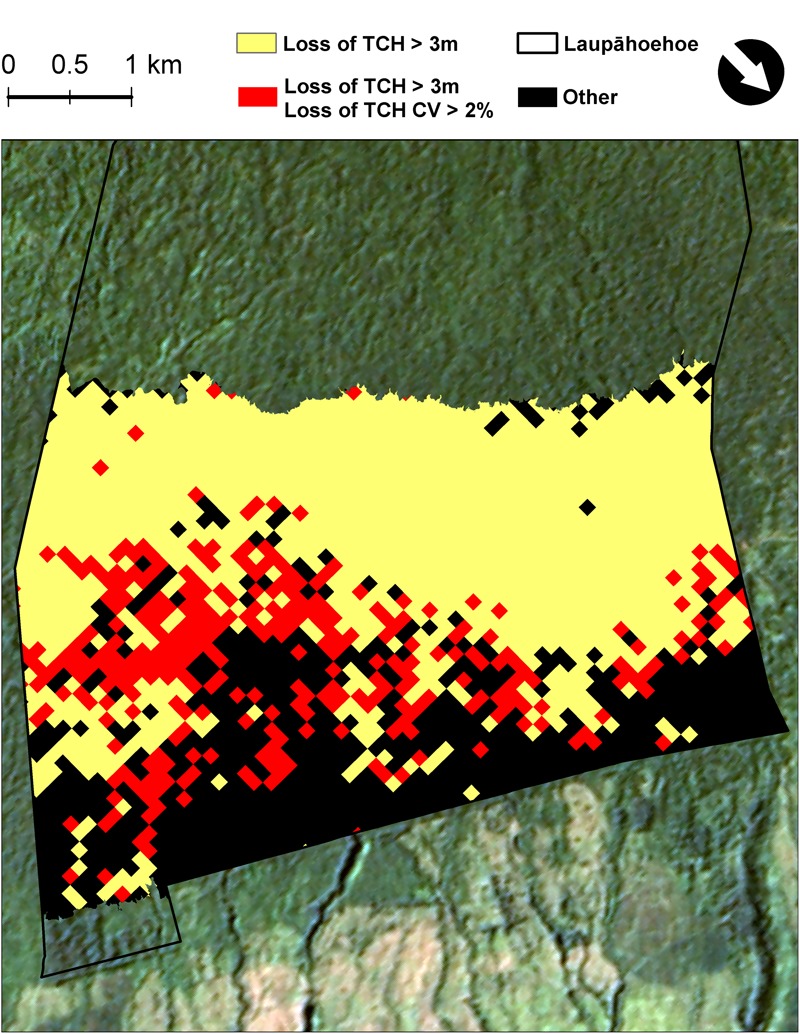
Map highlighting the transition zone (red) between highly invaded and native dominated forests, based on top-of-canopy height (TCH), decreasing and becoming less complex. Forests on a per hectare basis that have experienced a net loss in median TCH > 3 m (yellow) and a minimum decrease in TCH variation of 2% (red) from 2007 to 2016. Black areas did not experience a decrease in median TCH > 3 m.

Over the last decade in Laupāhoehoe, sub-montane forests have experienced a net loss in average canopy height, and therefore structure and aboveground carbon stock (**Figures 1, 3** and **Table 3**). The greatest losses were found in native-outbreak forests, with a median TCH loss of 5.4 m, equating to a mean loss of 60.2 Mg C ha^-1^ of ACD (33.4%). Established-invasion forests experienced the least amount of loss of only 1 m of height and 22.9 Mg C ha^-1^ of ACD (22.9%). However, established-invasion forests lost the most variation in canopy height distribution, with a decrease in the TCH coefficient of variation by 6.6% compared to a gain of 6.0% in native-outbreak forests. The transitional invaded-outbreak forests experienced a loss of median TCH (3.7 m), mean ACD of 41.3 Mg C ha^-1^ (27.8%) and loss of TCH variation (2.3%), as they have been shifting into monotypic stands of invasive tree species, particularly *P. cattleianum*. This transitional pattern of loss in TCH, in conjunction with a loss in TCH variation, is distinct and primarily found between invaded-outbreak and native-outbreak forests (**Figure 4**).

**Table 3:** Study region summary statistics for top-of-canopy height (TCH), coefficient of variation (CV), and aboveground carbon density (ACD).

Study landscape	2007 Mean TCH (m/SD)	2016 Mean TCH (m/SD)	2007 CV (%)	2016 CV (%)	2007 Mean ACD (Mg C ha^-1^/SD)	2016 Mean ACD (Mg C ha^-1^/SD)
Established-invasion	11.1 (5.5)	9.4 (4.1)	50.4	43.7	109.4 (53.1)	86.5 (38.5)
Invasion-outbreak	14.0 (6.4)	11.0 (4.8)	45.9	43.6	148.4 (40.7)	107.1 (28.9)
Native-outbreak	16.1 (6.7)	12.0 (5.7)	41.6	47.6	180.4 (54.2)	120.2 (42.3)

**FIGURE 5:**
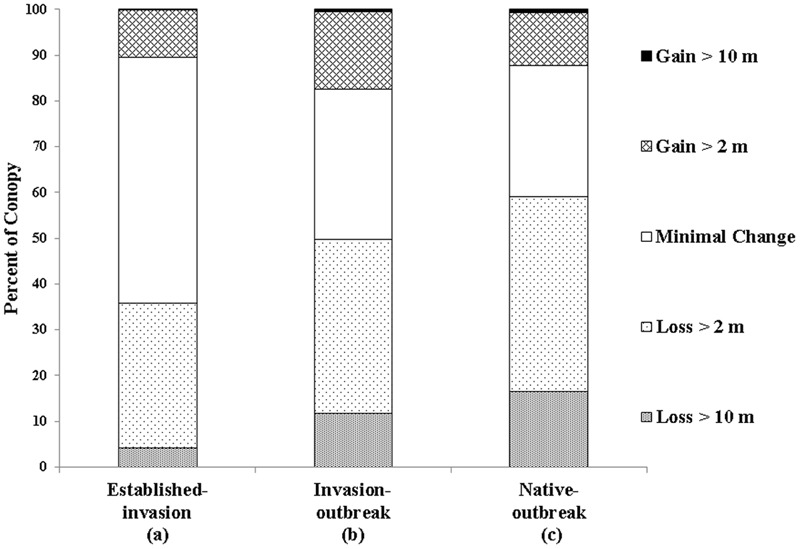
Net change in top-of-canopy height (TCH) between 2007 and 2016. Different letters below the *x*-axis indicate statistically significant differences between forest types (**Table 1**).

In all three forest types assessed, the majority of the TCH loss was in the 2–10 m class (established-invasion 32%, invasion-outbreak 38%, native-outbreak 43%; **Figure 5**), suggesting much of the loss was dieback or upper canopy breakage. However, there was also a considerable loss of canopy cover >10 m (upper canopy mortality), especially in the native-outbreak forests. Over 16% of the native-outbreak forest canopy had experienced a loss of TCH > 10 m compared to nearly 12 and 4% in invasion-outbreak and established-invasion forests.

Established-invasion forests experienced the lowest net change in TCH, with 54% of the canopy classified as minimal change (Δ TCH ± 2 m), compared to 33 and 29% for invasion-outbreak and native-outbreak forests, suggesting that the established-invasion forest canopies are at a more stable state in terms of TCH. All three forests underwent minimal (<1%) gains of trees taller than 10 m, or lateral growth of upper canopy trees into past gap spaces. Similar to TCH loss, the most TCH growth was observed in the 2–10 m class. Most of the TCH growth was observed in the invasion-outbreak forests with 17% of the canopy experiencing increases between 2 and 10 m, followed by native-outbreak (12%), and established-Invasion (10%).

**FIGURE 6:**
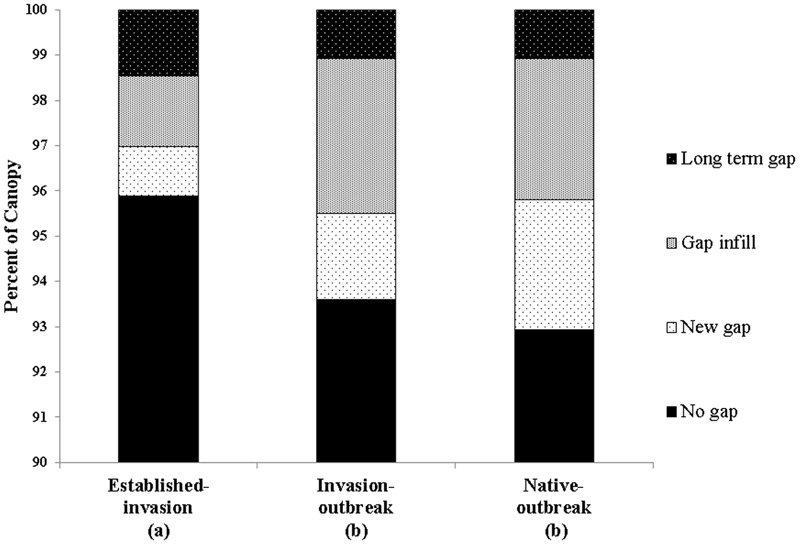
Change in gap dynamics between 2007 and 2016. Different letters below the *x*-axis indicate statistically significant differences between forest types (**Table 1**).

There was a net total of 7834 new gaps that formed between 2007 and 2016 in the three forest types assessed (**Table 4**). We found that established-invasion forests were the least dynamic in terms of net gap formation, with 97% of the canopy classified as no gaps or long-term gaps compared to invasion-outbreak (95%), and native-outbreak (94%; **Figure 6**). Established-invasion forest had fewer and smaller gaps, with 3.5 gaps ha^-1^, compared to invasion-outbreak, with 6.4 and native-outbreak with 8.7 gaps ha^-1^ (**Table 4**). When we accounted for the distribution of all gap dynamics (long-term, infill, new and no gaps) together within each forest type (**Figure 6**), a chi-square test revealed that established-invasion forests differed statistically from invasion-outbreak and native-outbreak forests (*p* < 0.05; Supplementary Table S3).

**Table 4:** Data table for dynamic gaps, gaps that have formed since 2007 and remained open in 2016.

Study landscape	Area (ha)	New Gaps	Gaps (ha^-1^)	Gap Size (m^2^) Mean/SD
Established-invasion	174.5	615	3.5	25.2 (29.9)
Invasion-outbreak	443.8	2833	6.4	26.5 (24.6)
Native-outbreak	504.3	4386	8.7	30.5 (34.2)

## Discussion

The upper canopy of native sub-montane forests within Laupāhoehoe are undergoing a structural shift, becoming shorter and less structurally diverse. Our spatially explicit results suggest that the most likely cause of the observed shift is a combined response to an episodic native insect outbreak, invasive species expansion, and climate change. These factors influencing the observed loss in canopy height were not uniform across the study landscape. In the upper elevations, dominated by native koa and ‘ōhi‘a trees (native-outbreak), it is clear that the 2013 koa moth outbreak resulted in stunted growth and substantial loss of upper canopy structure due to tree mortality. Our field observations, and a recent study by Banko et al. (2014), corroborate this assessment. Banko et al. (2014) found that the koa moth densities in Laupāhoehoe were the highest at 925 m and that the koa trees in the area experienced three large-scale defoliation events in the span of less than 1 year. They also noted that many of the mature koa trees were dead in Laupāhoehoe after the third defoliation event. Twenty-five weeks after the moth outbreak, Banko et al. (2014) also reported, in a nearby forest, that 18% of the koa trees were dead or unlikely to recover.

The combination of natural disturbance from the koa moth outbreak, potential drought stress, and presence of highly invasive tree species, created conditions that led to a moderate loss of canopy height (due to koa moth and drought) and complexity (due to invasive species) in the transition zone between highly invaded and native sub-montane forests (invaded-outbreak). The koa trees in the invaded-outbreak forests were also heavily defoliated (Banko et al., 2014). However, unlike the native-outbreak forests, invaded-outbreak forests contained higher densities of invasive tree species in closer proximity to the koa moth disturbance. The physiological traits prevalent in the invasive tree species, such as rapid growth rates and high nutrient content, would have given them a competitive advantage to fill space left by the dead koa trees. This could lead to a positive feedback and increased invasion, as seen in other parts of the island (e.g., Allison and Vitousek, 2004; Asner and Vitousek, 2005; Zimmerman et al., 2008).

The main factor shaping upper canopy structure in the highly invaded lower elevation forests (established-invasion) were the properties of the invasive species themselves. The koa moth outbreak had little to no effect on the structure of the highly invaded lower-elevation portions of the study landscape (established-invasion). Koa trees were scarce in established-invasion forests prior to 2013, and the area was outside the impact zone of the koa moth outbreak (800–2000 m elevation; Banko et al., 2014). Furthermore, the sharp increase in the density of TCH values between 8 and 12 m (**Figure 3A**), in the absence of a native mid-canopy layer, coincides with the average height of mature *P. cattleianum* stands observed in Hawai‘i with LiDAR (Asner et al., 2008). The observed lower number of gaps ha^-1^ in the established-invasion forests was unique and is indicative of the lower number of large trees and hence, tree falls capable of creating gaps with an area of >12 m^2^. Established-invasion forests provide a glimpse into the potential outcomes of increased upslope invasion, resulting in shorter statured, less dynamic forests.

High-resolution multi-temporal mapping of ACD provides a means to more accurately assess the current ACD stocks as well as predict potential shifts for future management. Forests are dynamic and management needs to be adaptive and proactive if we are to maintain the higher ACD seen in the native refugia forests of Hawaii and elsewhere. All three forest types experienced a loss of ACD over the 9 years period (**Table 3**). The largest loss was experienced in the native-outbreak forest. However, the native-outbreak forest also has the greatest potential to return to higher ACD values if native koa trees are able to reestablish before invasive species can expand into the openings left by the kao moth outbreak. The smaller loss of ACD in the Established-invasion forests is not surprising; most of the ACD loss to these forests was due to invasive species such as *P. cattleianum* and *F. rubiginosa* replacing the native forest structure decades ago (Asner et al., 2009; Jones, 2011). The Invasion-outbreak forests appear to be at a higher risk compared to Native-outbreak forests for permanent ACD loss. This risk of permanent ACD loss is the combined influence of natural disturbances and proximity to invasive species.

Although the pattern of loss in Hawaiian sub-montane forests is clear, the degree to which the potential factors (climate, invasive species, and insect outbreak) covary or individually influence the observed pattern cannot be tested by this study, and warrants future experimental work. A caveat in the case of dynamic gaps is that the number of gaps does not include any gap that forms after 2007 and infills by 2016. This may have a stronger influence in the established-invasion forests. Despite the greater potential for some gaps to go uncounted, the reduced number of larger trees in these forests, and the monotypic stands of *P. cattleianum* in 2007 make the probability of gaps with an area > 12 m^2^ forming and infilling between 2007 and 2016 low.

Tracking the loss of upper canopy height and variation with LiDAR can be used as a cost-effective way to assess forest canopy condition. This approach is particularly useful for identifying and tracking invasive species through time. Most of the highly invaded low-elevation forests in Hawai‘i have crossed management thresholds into novel ecosystems, where restoration to native ecosystems at a landscape scale is not feasible (Mascaro et al., 2012). As the invasion front further perforates upslope into sub-montane forests during episodic canopy dieback (natural or otherwise), it is critical to track where the invasion is occurring, its rate, and how it is changing forest structure and function. The results of this study can provide both a simple framework and a baseline for monitoring the location and rate of canopy structural change in Hawaiian sub-montane forests and elsewhere. With a recent report of a new tree pathogen, *Ceratocystis fimbriata*, selectively killing large numbers of native ‘ōhi‘a trees (Keith et al., 2015), and future koa moth outbreaks, there is a need for early detection of ongoing structural changes to native Hawaiian forests if target conservation measures are desired before the sub-montane forests are lost to invasive alien species.

## Author Contributions

CB designed the study, performed data analysis and wrote the manuscript. GA designed the study, performed data analysis, wrote the manuscript, acquired the funding, and collected the remote sensing data.

## Conflict of Interest Statement

The authors declare that the research was conducted in the absence of any commercial or financial relationships that could be construed as a potential conflict of interest.

## Supplementary Material

FIGURE S1 United States drought monitor graph from January 2001 to January 2017 for Hawaii county, highlighting the 2008–2014 drought. Drought classification criteria can be found in Supplementary Table S4. Data obtained from http://droughtmonitor.unl.edu.

FIGURE S2 Mean annual precipitation (MAP) for the study landscape. Data obtained from the Rainfall Atlas of Hawaii http://climate.geography.hawaii.edu/ (Giambelluca et al., 2013). Data was only available up to 2012. The red verticle line denotes the start of the 2008–2014 drought. The solid black horizontal line is the study landscape MAP for the Hawaii Rainfall Atlas 30-years base period (1978–2007). The dashed horizontal lines are one standard deviation from the mean of the base period.
