# Supplementary figures and images for: Episodic Canopy Structural Transformations and Biological Invasion in a Hawaiian Forest
Source: Front Plant Sci. 2017 Jul 21;8:1256. doi: 10.3389/fpls.2017.01256 (PMC5519564; doi:10.3389/fpls.2017.01256)

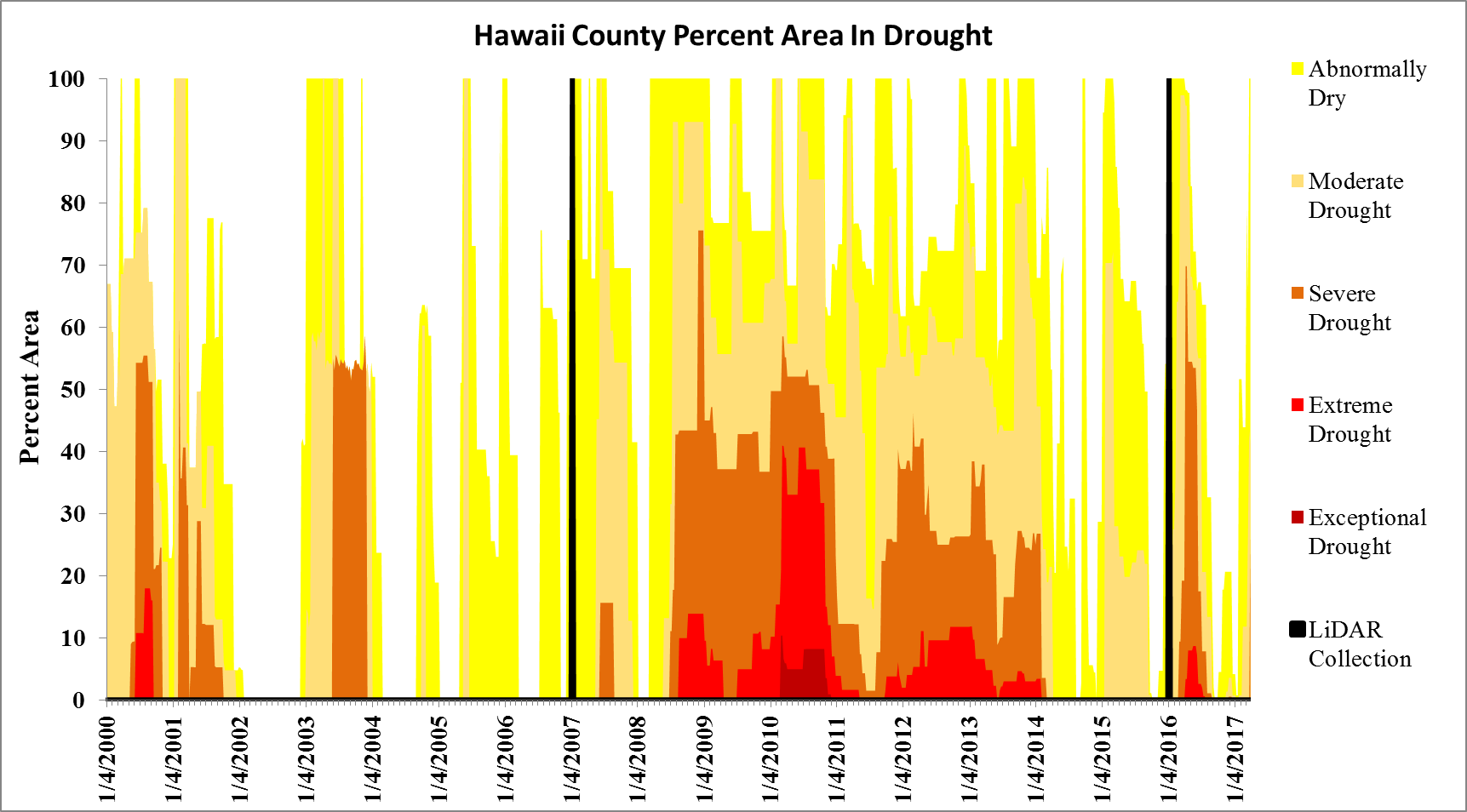

Supplement: FIGURE S1 — United States drought monitor graph from January 2001 to January 2017 for Hawaii county, highlighting the 2008–2014 drought. Drought classification criteria can be found in Supplementary Table S4. Data obtained from http://droughtmonitor.unl.edu. [file Image_1.TIF]

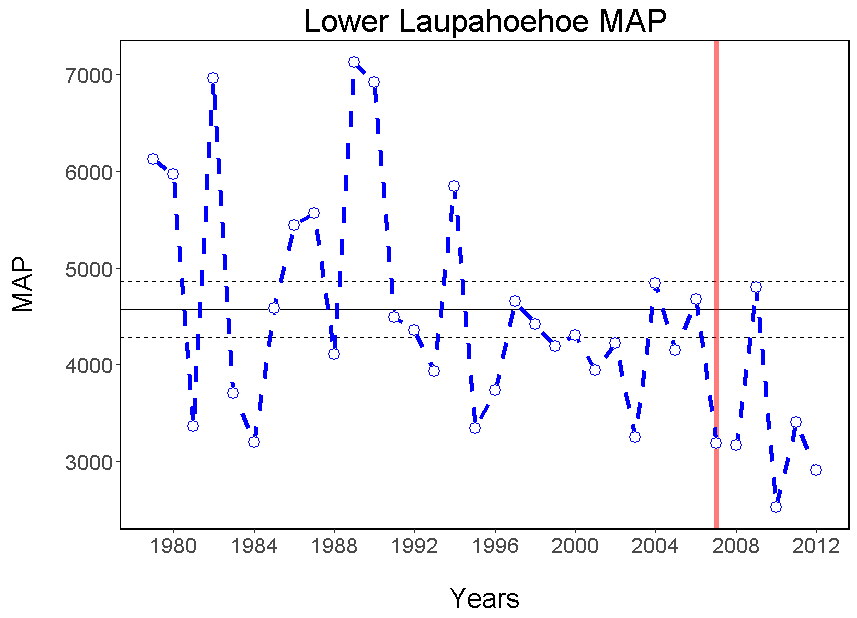

Supplement: FIGURE S2 — Mean annual precipitation (MAP) for the study landscape. Data obtained from the Rainfall Atlas of Hawaii http://climate.geography.hawaii.edu/ (Giambelluca et al., 2013). Data was only available up to 2012. The red verticle line denotes the start of the 2008–2014 drought. The solid black horizontal line is the study landscape MAP for the Hawaii Rainfall Atlas 30-years base period (1978–2007). The dashed horizontal lines are one standard deviation from the mean of the base period. [file Image_2.TIFF]
