# Supplementary material for: Episodic Canopy Structural Transformations and Biological Invasion in a Hawaiian Forest
Source: Front Plant Sci. 2017 Jul 21;8:1256. doi: 10.3389/fpls.2017.01256 (PMC5519564; doi:10.3389/fpls.2017.01256)
Supplement: Supplementary file 3 [file Table_1.DOCX]

**Supplementary Material**

**Supplementary Table 1**: *Psidium cattleianum* stem density by elevation within Laupāhoehoe from Jones (2011).

| Elevation  (m) | *Psidium cattleianum* stem density  (trees ha ^-1^) |
| --- | --- |
| 767 | 9130 |
| 778 | 8750 |
| 791 | 5250 |
| 804 | 6630 |
| 818 | 6380 |
| 834 | 5130 |
| 852 | 3880 |
| 864 | 2750 |
| 874 | 3250 |
| 884 | 630 |
| 898 | 1250 |
| 912 | 1130 |
| 926 | 380 |
| 938 | 250 |
| 950 | 130 |
| 962 | 500 |
| 971 | 0 |
