# Supplementary material for: Episodic Canopy Structural Transformations and Biological Invasion in a Hawaiian Forest
Source: Front Plant Sci. 2017 Jul 21;8:1256. doi: 10.3389/fpls.2017.01256 (PMC5519564; doi:10.3389/fpls.2017.01256)
Supplement: Supplementary file 4 [file Table_2.DOCX]

**Supplementary Table 2:** *Acacia koa* stem density by elevation within Laupāhoehoe from Scowcroft and Sakai (1984).

| Elevation  (m) | *Acacia koa* stem density  (trees ha ^-1^) |
| --- | --- |
| 770 | 840 |
| 810 | 890 |
| 840 | 940 |
| 890 | 820 |
| 920 | 2840 |
| 980 | 2200 |
| 1090 | 1660 |
| 1110 | 3340 |
| 1260 | 2200 |
| 1330 | 4990 |
