# Supplementary material for: Episodic Canopy Structural Transformations and Biological Invasion in a Hawaiian Forest
Source: Front Plant Sci. 2017 Jul 21;8:1256. doi: 10.3389/fpls.2017.01256 (PMC5519564; doi:10.3389/fpls.2017.01256)
Supplement: Supplementary file 5 [file Table_3.DOCX]

**Supplementary Table 3**: Chi-square analysis of gap dynamics and change in top-of-canopy height (TCH) by class. Numbers in bold are significantly different at *p* < 0.05.

|  |  | |  | |
| --- | --- | --- | --- | --- |
| **Landscape Comparisons** | **Gap Distribution** | | **Δ TCH** | |
|  | **raw *p*** | **adj *p*** | **raw p** | **adj p** |
| Established-invasion vs. Invasion-outbreak | **0.0147** | **0.0221** | **< 0.001** | **< 0.001** |
| Established-invasion vs. Native-outbreak | **0.0013** | **0.0038** | **< 0.001** | **< 0.001** |
| Invasion-outbreak vs. Native-outbreak | 0.2307 | 0.2307 | **< 0.001** | **< 0.001** |
