# Supplementary material for: Episodic Canopy Structural Transformations and Biological Invasion in a Hawaiian Forest
Source: Front Plant Sci. 2017 Jul 21;8:1256. doi: 10.3389/fpls.2017.01256 (PMC5519564; doi:10.3389/fpls.2017.01256)
Supplement: Supplementary file 6 [file Table_4.DOCX]

**Supplementary Table 4:** Drought severity classification criteria for Supplementary Figure 1 obtained from the U.S. Drought Monitor (<http://droughtmonitor.unl.edu>).

| Name | Palmer Drought  Severity Index  (PDSI) | CPC Soil Moisture  Model (Percentiles) | USGS Weekly  Streamflow  (Percentiles) | Standardized  Precipitation Index  (SPI) | Objective Drought  Indicator Blends  (Percentiles) |
| --- | --- | --- | --- | --- | --- |
| Abnormally Dry | -1.0 to -1.9 | 21 to 30 | 21 to 30 | -0.5 to -0.7 | 21 to 30 |
| Moderate Drought | -2.0 to -2.9 | 11 to 20 | 11 to 20 | -0.8 to -1.2 | 11 to 20 |
| Severe Drought | -3.0 to -3.9 | 6 to 10 | 6 to 10 | -1.3 to -1.5 | 6 to 10 |
| Extreme Drought | -4.0 to -4.9 | 3 to 5 | 3 to 5 | -1.6 to -1.9 | 3 to 5 |
| Exceptional Drought | -5.0 or less | 0 to 2 | 0 to 2 | -2.0 or less | 0 to 2 |
